# Contrasting effects of organic materials versus their derived biochars on maize growth, soil properties and bacterial community in two type soils

**DOI:** 10.3389/fmicb.2023.1174921

**Published:** 2023-05-25

**Authors:** Xiaosong Yue, Xing Liu, Fei Wang, Changwei Shen, Ying Zhang

**Affiliations:** Henan Engineering Research Center of Biological Pesticide & Fertilizer Development and Synergistic Application, College of Resources and Environmental Sciences, Henan Institute of Science and Technology, Xinxiang, China

**Keywords:** organic material, biochar, soil properties, soil enzyme activity, soil bacterial community

## Abstract

The objective of this study was to assess the benefit of applying biochar instead of its feedstock in enhancing soil quality. To accomplish this, we investigated the short-term effects of two organic materials and their derived biochars on maize growth, soil properties, and microbial community in fluvo-aquic and red soil with a pot experiment. Five treatments were applied to each soil, namely, the addition of straw, manure, straw-derived biochar, manure-derived biochar, and the control with no addition of any organic materials and biochar. Our results revealed that straw decreased the shoot biomass of maize in both soils, while straw-derived biochar, manure and manure-derived biochar increased it by 51.50, 35.47 and 74.95% in fluvo-aquic soil and by 36.38, 117.57 and 67.05% in red soil compared with the control, respectively. Regarding soil properties, although all treatments increased soil total organic carbon, straw and manure exhibited more pronounced effects on improving permanganate-oxidizable carbon, basal respiration, and enzyme activity compared with their derived biochars. Manure and its biochar had more significant effects on improving soil available phosphorus, whereas straw and its biochar exhibited more ameliorating effects on available potassium. Straw and manure consistently decreased bacterial alpha diversity (Chao1 and Shannon index) and altered bacterial community composition in the two soils by increasing the relative abundances of Proteobacteria, Firmicutes, and Bacteroidota and decreasing those of Actinobacteriota, Chloroflexi, and Acidobacteriota. More specifically, straw had a greater effect on Proteobacteria, whereas manure affected Firmicutes more. While straw-derived biochar had no effect on bacterial diversity and bacterial community composition in both soils, manure-derived biochar increased bacterial diversity in the fluvo-aquic soil and altered bacterial community composition in the red soil by increasing the relative abundances of Proteobacteria and Bacteroidota and decreasing that of Firmicutes. In summary, owing to the input of active organic carbon, straw and manure exhibited more pronounced short-term effects on soil enzyme activity and bacterial community compared with their derived biochar. Furthermore, straw-derived biochar was found to be a better option than straw in promoting maize growth and nutrient resorption, while the choice of manure and its biochar should be determined by the soil type.

## 1. Introduction

China is one of the largest agricultural countries in the world, with less than 9% of the world’s cultivated land feeding nearly 20% of the world’s population (Liu Z. J. et al., 2021). Such outstanding achievement is largely ascribable to the use of chemical fertilizers in China (Zhu and Jin, 2013). However, the unscientific use of chemical fertilizers and ignorance concerning organic and microbial inputs have resulted in soil fertility degradation, such as acidification, salinization, nutrient imbalance, and microecological disorders (Guo et al., 2010; Gupta et al., 2022). Concomitantly, the organic matter of cropland soils decreases with intensive agricultural management. Hence, a fundamental shift toward agricultural green development is required to ensure sustainable food security and protect the ecological environment (Davies and Shen, 2020).

The canonical practices used to reverse soil fertility, particularly organic matter, are straw returning and organic fertilization. The former has been demonstrated to reduce soil bulk density, increase porosity, enhance the available nutrient content, and promote soil organic carbon storage and stability, thereby improving crop productivity (Zhu et al., 2015). Furthermore, straw returning was found to improve soil microbial richness and diversity (Sun et al., 2015). These benefits have also been observed for organic fertilization (Ren et al., 2018; Rayne and Aula, 2020). Moreover, Sun et al. (2015) noted that organic fertilization exerted a more positive influence on soil microbial diversity than straw returning. In addition, straw returning has a few disadvantages. First, it decreases the soil water content and temperature and, as a result, declines the seedling emergence rate (Zhao J. L. et al., 2019). Second, straw incorporation can lead to the deficiency of the soil available nitrogen because of the high carbon-to-nitrogen ratio of straw, and aggravate the competition for nitrogen between crop and soil microbes and finally negatively affect crop growth and yield (Li et al., 2016). Third, though straw returning improves soil physicochemical properties, such as water-storage capacity and porosity, it also provides a more suitable living environment favoring various pathogens and insect eggs, which aggravates crop diseases and insect pests (Liu T. et al., 2016). In addition, straw returning increases the emission of greenhouse gases such as carbon dioxide (Liu et al., 2014). Compared with straw returning, manure is a type of traditional fertilizer with a lower carbon-to-nitrogen ratio and may exhibit better fertilizer efficiency, while it can contribute to greenhouse gas emissions in different treatment processes, such as manure storage, fermentation, and application to soil (Chadwick et al., 2011). Inadequately treated manure used as fertilizer is more likely to carry harmful substances such as pathogens and parasite eggs (Wan et al., 2020).

Biochar is a type of solid multifunctional material with rich carbon, developed pore structure, huge specific surface area, rich oxygen-containing functional groups, high aromatization, and stable properties. It is produced by high-temperature pyrolysis (usually <700°C) of biomass materials from agricultural, forestry, and animal husbandry wastes under anaerobic conditions such as straw, litter, livestock manure, and other biomass materials (Ahmad et al., 2014; Chen et al., 2019). Compared with direct straw returning, biochar can better reduce soil bulk density, increase soil permeability, improve soil buffering function, enhance soil water and nutrient retention capacity, adjust soil acid–base balance, promote crop root elongation and growth, and prevent issues such as a low seedling emergence rate and yield decline (Tan et al., 2017). Because of the high-temperature treatment of biochar, the parasite eggs and pathogens possibly carried by the biomass materials are completely killed, which avoids the risk of diseases and insect pests at the later stage of crop growth. Notably, previous studies have shown that the mean residence time of biochar in soil is approximately 2,000 years, while the half-life is approximately 1,400 years (Kuzyakov et al., 2009). As a beneficial soil amendment, biochar has recently gained increasing attention in modern agriculture (Tan et al., 2017; Chen et al., 2019; Palansooriya et al., 2019).

As the biogeochemical cycle and material metabolism of elements in the soil are driven by soil microbes, the composition of soil microbes is directly related to soil fertility and crop productivity. Furthermore, soil biological stability is largely affected by the soil microbial community structure, which is crucial to the stability of the terrestrial ecosystem (Griffiths and Philippot, 2013). As a strategy to enhance soil fertility, organic amendments strongly influence soil microbial community directly by their own and indirectly through changing soil physico-chemical properties, while different organic amendments have different mechanisms of action. Straw and manure are rich in dissolved organic carbon, which is the organic carbon source for microorganisms present in the soil. Thus, straw and manure exhibit rapid changes in the microbial community composition. However, the long-term effects are gradually weakened due to the consumption of dissolved organic carbon. Ros et al. (2006) noted that microbial communities did not show major changes after soil was amended with bio waste, green waste, manure, and sewage sludge after more than a decade. Biochar shows substantial short- and long-term effects on soil microbial communities by space for colonization and changing soil conditions (e.g., moisture and pH). It can persist in the soil for a long period as it contains a large amount of stable carbon that is resistant to decay. The porous structure of biochar can adsorb nutrients and water in soil and provide a good habitat for soil microbes (Palansooriya et al., 2019). This could explain why straw biochar exhibited more pronounced effect on the abundance and diversity of bacteria compared with straw in a field experiment carried out for four consecutive years (Li et al., 2020). Therefore, to comprehensively evaluate the various positive effects of biochar on the soil properties and microbes compared with its raw material, it is necessary to undertake a shorter-term study. This is especially important for crops with short growing period, such as summer maize.

Furthermore, it has been proved that biochar can enhance soil health and alter the composition and structure of the soil microbial community (Sun et al., 2016; Sheng and Zhu, 2018). However, different results have also been reported. Liao et al. (2021) found that biochar amendment had limited impacts on rhizosphere bacterial community composition in alkaline calcareous soils. Zhang et al. (2019) also demonstrated that biochar could alter the bacterial communities in acidic soil but not alkaline soil. The differences in soil types tested and feedstocks could be the prime reasons for these discrepancies (Li et al., 2020). It is equally necessary to assess the effects of biochar with different feedstocks on soil fertility and microbial diversity, particularly in different types of soil. In this study, alkaline fluvo-aquic soil and acid red soil were selected as the test soils to explore the short-term effects of two highly disparate and widely sourced biomass materials, namely, wheat straw and swine manure, and their derived biochars on maize growth, soil properties, and microbial diversity. We hypothesized that (1) straw and manure can more rapidly alter soil bacterial community and enzyme activities rather than their derived biochar in short term and (2) the response of maize growth, soil chemical and biological characteristics to biochar input was positive, but the beneficial effect of biochar should be dependent on its feedstocks.

## 2. Materials and methods

### 2.1. Materials

Two type soils were selected for this experiment. The fluvo-aquic soil was collected from Xinxiang, Henan province (35.4°N, 114.4°E), the red soil was collected from Sanming, Fujian province (26.8°N, 116.8°E). Soil samples were collected from the surface (0–20 cm) and hand-picked to remove obvious plant debris, air-dried, ground, and sieved through a 2 mm sieve, and then reserved for the pot experiment. The fluvo-aquic soil properties were as follows: 9.93 g kg^−1^ total organic carbon, 75.25 mg kg^−1^ available N, 16.25 mg kg^−1^ available P, 186.30 mg kg^−1^ available K and a soil pH of 8.21. The red soil properties were as follows: 11.27 g kg^−1^ total organic carbon, 114.33 mg kg^−1^ available N, 15.91 mg kg^−1^ available P, 95.20 mg kg^−1^ available K and a soil pH of 4.55.

Two types of biochar were prepared from wheat straw and swine manure, which named as SBC and MBC, respectively. The preparation of biochar was as follows: first, the two biomass materials were oven dried and pulverized with a 1 mm sieve, and then the powder was compacted in ceramic crucible equipped with a cover and pyrolyzed for 4 h at 550°C in a muffle furnace under oxygen-limited condition, naturally cooled it to room temperature, and bag it for later use. The chemical properties of the biomass material and biochar listed in Supplementary Table S1.

### 2.2. Experimental design

A pot experiment was conducted in natural condition to study the effects of biomass materials and their derived biochar to maize growth, soil nutrients and microbial diversity. Each pot (10 cm height and 9 cm diameter) was filled with 200 g soil and was fertilized with the following amounts of macronutrients: N 150, P_2_O_5_ 100, K_2_O 100 mg kg^−1^ soil supplied with NH_4_NO_3_, KH_2_PO_4_, K_2_SO_4_, respectively. There were five treatments for each soil, including the application of wheat straw (S), swine manure (M), wheat straw-derived biochar (SBC), swine manure-derived biochar (MBC) and the control with no addition of any organic materials and biochar (CK), the addition amount of two types of biochar was 2 g per pot at a rate of 1% (w/w), the addition rate of wheat straw or swine manure was calculated according to biochar addition amount divided by each biochar production rate, with the addition amount of wheat straw (biochar production rate: 27%) being 7.41 g per pot and swine manure being (biochar production rate: 37%) 5.41 g per pot. The treatments in fluvo-aquic soil and red soil were referred to as F_CK, F_S, F_SBC, F_M, F_MBC, R_CK, R_S, R_SBC, R_M, R_MBC, respectively. Each treatment was performed in four repeats (pots).

The experiment was arranged on April, 2021 at Henan Institute of Science and Technology (35.3°N, 113.9°E), Xinxiang, China, in a rain-sheltered wire house under open-air conditions. After all fertilizers were evenly mixed with the soil, three maize seeds were sown per pot and thinned to one per pot after seedling emergence, all treatments were managed consistently. The experiment was finished 45 days after emergence of maize. At the end of the experiment, both soil and plant samples were collected. The plants were carefully taken out from soils, The plant samples were washed and separated into roots and shoots, dry weight of each part was weighed, and then crushed for nutrient element analysis. After removing the plants, the soil remaining in each pot was mixed well and split into three subsamples for subsequent analysis. One was stored at 4°C for soil respiration and enzymatic activity, one was air-dried and stored for soil physicochemical properties analysis, and one subsample was stored at −80°C for molecular ecological assays.

### 2.3. Analysis of soil physicochemical properties

Air-dried soil samples were triturated with a wooden roller, passed through a sieve of 1 mm for soil pH, available N, available P and available K analysis, and passed through a sieve of 0.15 mm for soil total organic carbon analysis. The experimental parameters were measured according to the soil physicochemical analysis handbook (Bao, 2008). Soil total organic carbon (TOC) was measured using the chromic and titration procedure. The soil pH was determined potentiometrically in 1:2.5 soil/distilled water suspensions after shaking. The soil available N (AN) was determined using alkaline hydrolysis diffusion, the soil available P (AP) was determined using Olsen’s method, and the soil available K (AK) was extracted with 1 mol L^−1^ of ammonium acetate and determined using a flame photometer. Soil permanganate oxidizable carbon (POXC) was determined by the method as the description of Lefroy et al. (1993).

### 2.4. Analysis of soil enzymatic activities and soil basal respiration

The fresh soil samples stored in 4°C were used for analysis of soil enzymatic activities and soil basal respiration (SBR). Soil basal respiration (SBR) referenced our previous study described and slight changed (Zhang et al., 2015). Briefly, the 20 g fresh soil was incubated with 10 mL of 0.1 M NaOH for 24 h at 37°C to absorb the CO2, and then the residual alkali was titrated with standardized HCl. The activities of several soil enzymes, including urease (UA), sucrase (SU), catalase (CA) and β-glucosidase (GLU), were determined according to the textbook edited by Li et al. (2008). The mean soil enzyme (GMea) activity was calculated based on the geometric mean of all tested enzymes (Zhang et al., 2015), the formula is given as:

$$\mathrm{GMea}={\left(\mathrm{urease}\times \mathrm{sucrase}\times \mathrm{catalase}\times \beta -\mathrm{glucosidase}\right)}^{1/4}.$$

### 2.5. Soil DNA extraction, high-throughput sequencing and bioinformatic analysis

Total microbial genomic DNA of each soils was extracted using the E.Z.N.A.® soil DNA Kit (Omega Bio-tek, Norcross, GA, United States) according to manufacturer’s instructions. The 1.0% agarose gel electrophoresis and a NanoDrop® ND-2000 spectrophotometer (Thermo Scientific Inc., United States) were used to determine the concentration and quality of DNA. The hypervariable region V3-V4 of the bacterial 16S rRNA gene were amplified with primer pairs 338F (5’-ACTCCTACGGGAGGCAGCAG-3′) and 806R (5’-GGACTACHVGGGTWTCTAAT-3′) by an ABI GeneAmp® 9,700 PCR thermocycler (ABI, CA, United States) (Liu C. et al., 2016). The PCR reaction mixture including 4 μL 5 × Fast Pfu buffer, 2 μL 2.5 mM dNTPs, 0.8 μL each primer (5 μM), 0.4 μL Fast Pfu polymerase, 10 ng of template DNA, and ddH_2_O to a final volume of 20 μL. Amplification conditions for PCR were as follows: initial denaturation at 95°C for 3 min, followed by 27 cycles of denaturing at 95°C for 30 s, annealing at 55°C for 30 s and extension at 72°C for 45 s, and single extension at 72°C for 10 min, and end at 10°C. Triplicate amplifications were performed on all samples. The PCR product was extracted from 2% agarose gel and purified using the AxyPrep DNA Gel Extraction Kit (Axygen Biosciences, Union City, CA, USA) according to manufacturer’s instructions and quantified using Quantus™ Fluorometer (Promega, USA).

Purified amplicons were pooled in equimolar amounts and paired-end sequenced on an Illumina MiSeq PE300 platform (Illumina, San Diego, United States) according to the standard protocols by Majorbio Bio-Pharm Technology Co. Ltd. (Shanghai, China). The raw sequencing reads were deposited into the NCBI Sequence Read Archive (SRA) database (Accession Number: PRJNA888047).

The biological analysis process was as follows: after the sample separation of the PE reads obtained by MiSeq sequencing, the quality control and filtering of the double-terminal Reads were carried out according to the sequencing quality, and the optimized data after the quality control splicing was obtained by splicing according to the overlap relationship between the two-terminal Reads. Then the sequence denoising method (DADA2) was used to process the optimized data to obtain the representative sequence and abundance information of ASV (Amplicon Sequence Variants). Based on the representative sequence and abundance information of ASV, a series of statistical or visual analysis could be carried out, such as species taxonomy analysis, community diversity analysis, species difference analysis, correlation analysis and phylogenetic analysis. The structural equation model (SEM) was constructed to assess how soil properties and basal respiration directly and indirectly affected soil bacterial diversity and enzyme activities in two soils. The model assumes was run by the AMOS 18.0 software (IBM, Chicago, IL, United States). Adequate model fits were indicated by the *χ*^2^ test (*p* > 0.05), goodness-of-fit index (GFI), and a low root-mean-square error of approximation (RMSEA) (< 0.001) (Hooper et al., 2008).

### 2.6. Statistical analysis

The significant differences between treatments were determined using one-way ANOVA (Duncan’s multiple comparisons at 95% confidence level). Mean values ± standard deviations were reported in this study. Bioinformatic analysis of the soil microbiota was carried out using the Majorbio Cloud platform.^1^ Based on the ASVs information, alpha diversity indices including Chao1 richness and Shannon index were calculated with Mothur v1.30.1 (Schloss et al., 2009). The similarity among the microbial communities in different samples was determined by principal coordinate analysis (PCoA) based on Bray–curtis dissimilarity using Vegan v2.5–3 package. The PERMANOVA test was used to assess the percentage of variation explained by the treatment along with its statistical significance using Vegan v2.5–3 package. The distance-based redundancy analysis (db-RDA) was performed using Vegan v2.5–3 package to investigate effect of soil physicochemical properties on soil bacterial community structure. Forward selection was based on Monte Carlo permutation tests (permutations = 9,999). Values of the *x*- and *y*-axis and the length of the corresponding arrows represented the importance of each soil physicochemical properties in explaining the distribution of taxon across communities.

## 3. Results

### 3.1. Plant biomass and accumulation of nitrogen, phosphorus, and potassium

**Figure 1:**
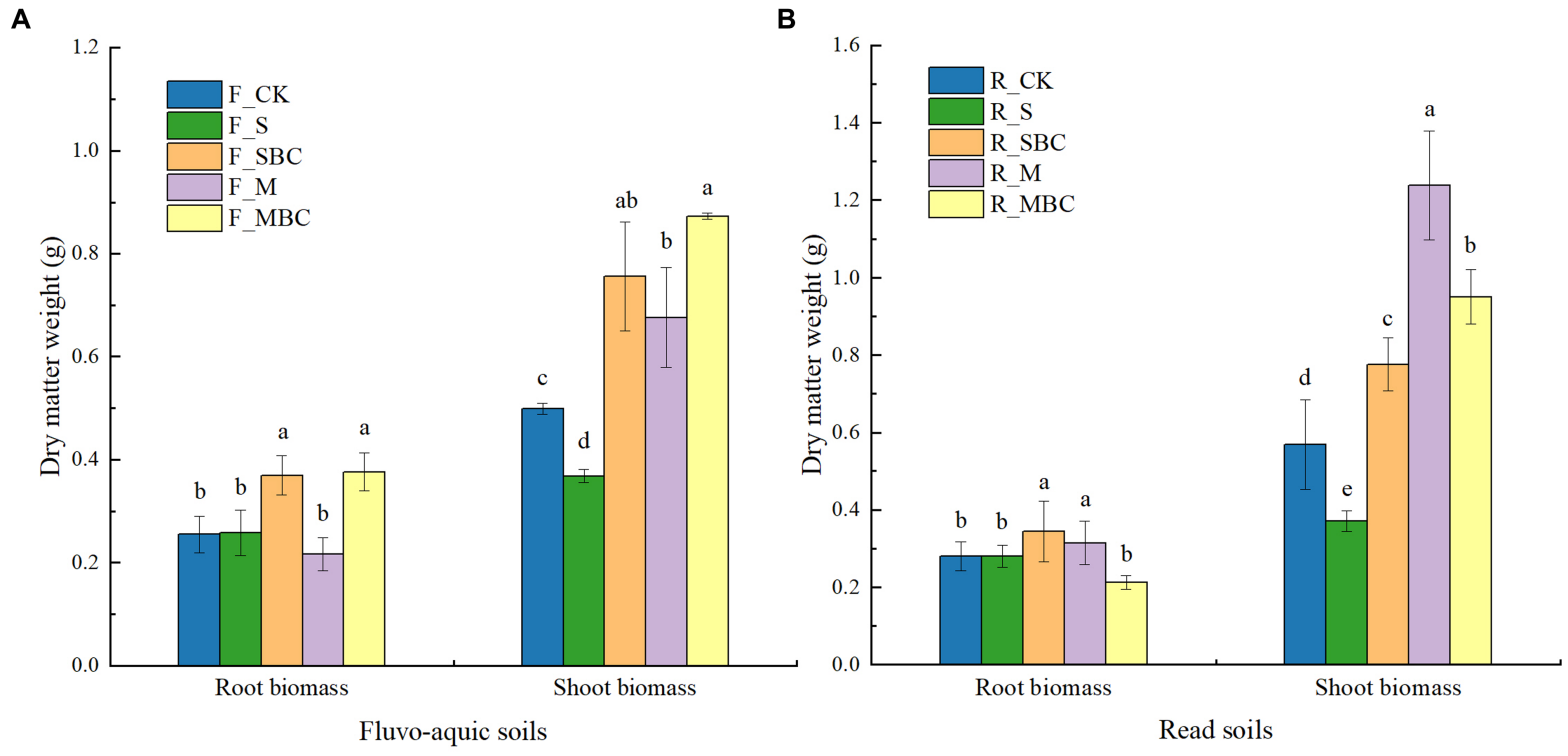
Short-term effects of organic materials and their derived biochars on maize root and shoot biomass in fluvo-aquic soil **(A)** and red soil **(B)**. Data in the figure are represented by mean ± SD (*n* = 4). Error bars indicate standard deviation. The different lowercase letters on error bars represent significant differences (*p* < 0.05).

Plant biomass under different treatments were listed in Figure 1. In fluvo-aquic soil, the root biomass of maize in F_SBC and F_MBC signifcantly increased by 44.90 and 47.65% respectively, compared with F_CK. For the red soil, maize root biomass in R_SBC and R_M signifcantly increased by 22.42 and 11.98%, respectively, compared with R_CK. The shoot biomass of maize in SBC, M and MBC were higher by 51.50, 35.47 and 74.95% in fluvo-aquic soil and by 36.38, 117.57 and 67.05% in red soil compared with CK. Conversely, treatment with S significantly reduced the shoot biomass of maize in two soils. In addition, the root and shoot biomass treated by SBC were higher than S in two soils. For manure and its biochar, MBC showed superiority over M in fluvo-aquic soil, while the opposite situation was observed in red soil.

**Figure 2:**
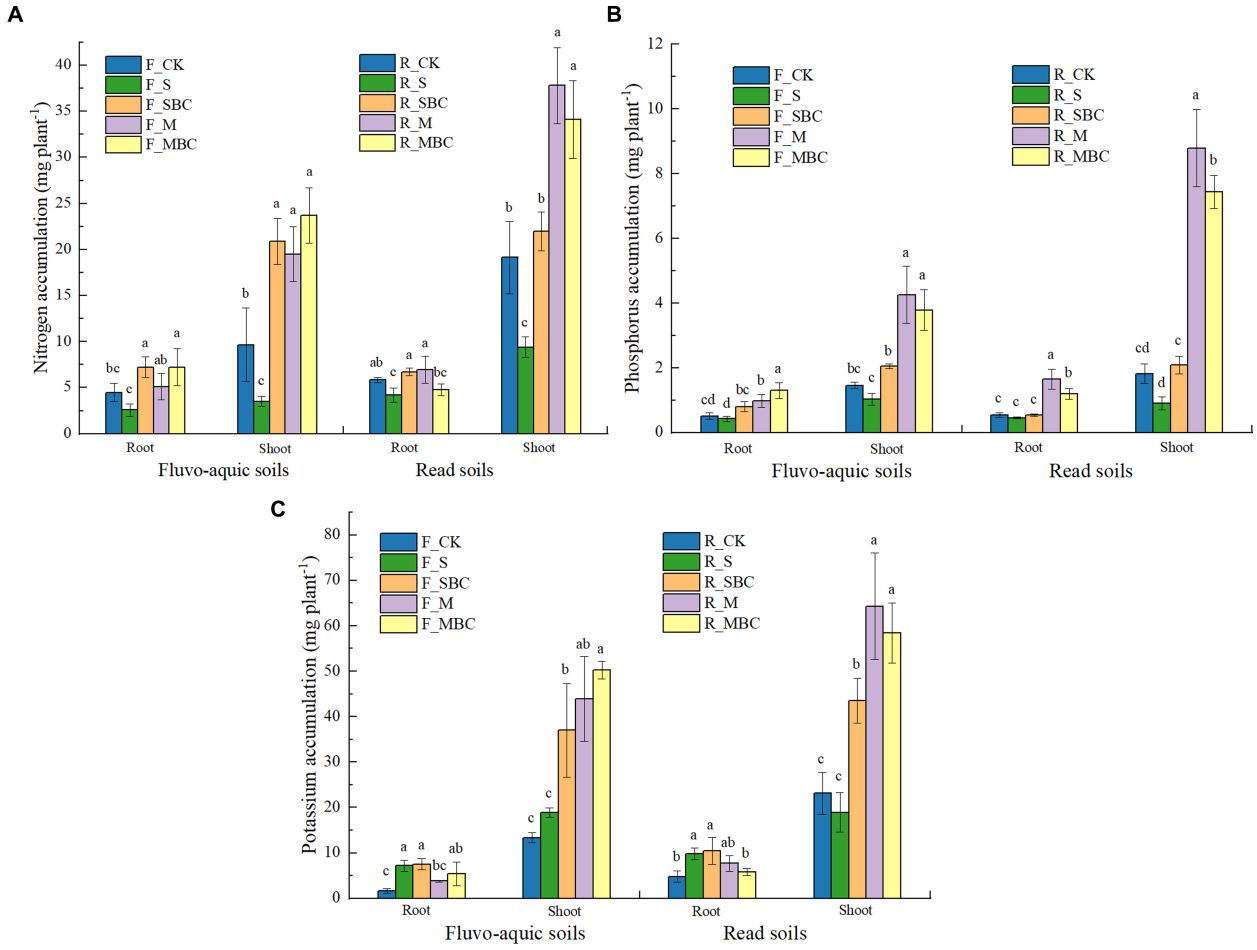
Short-term effects of organic materials and their derived biochars on maize root and shoot nitrogen **(A)**, phosphorus **(B)**, and potassium **(C)** accumulation. Data in the figure are represented by mean ± SD (*n* = 4). Error bars indicate standard deviation. The different lowercase letters on error bars represent significant differences (*p* < 0.05).

In terms of nutrient accumulation in maize plants, compared to CK, the shoot nitrogen accumulation was increased by F_SBC, F_M and F_MBC in fluvo-aquic soil, and increased by R_M and R_MBC in red soil (Figure 2A). The root and shoot phosphorous accumulation were increased by M and MBC in two soils compared with CK, while the effect of S and SBC treatment were not significant (Figure 2B). Moreover, the root potassium accumulation was increased by S and SBC, and the shoot potassium accumulation was increased by SBC, M, and MBC in two soils, compared with CK.

### 3.2. Soil physicochemical properties and enzyme activities

Soil physicochemical properties under different treatments were listed in Table 1. Soil pH was decreased by F_S and F_M compare to CK in fluvo-aquic soil, while it was increased by R_SBC, R_M and R_MBC in red soil. The TOC of the two soils were increased by all the treatments, while the POXC were increased by F_S, F_M, and F_MBC in the fluvo-aquic soil and increased by R_S and R_M in the red soil, compared with CK. Furthermore, F_S and F_M could increase the AN in the fluvo-aquic soils, while R_S, R_SBC, and R_MBC decreased in the red soil. M and MBC could increase the AP of the two soils, while S and SBC had stronger promotion on soil AK.

**Table 1:** Soil physiochemical properties in different treatments.

Treatment	pH	TOC	POXC	AN	AP	AK
		g kg^−1^	g kg^−1^	mg kg^−1^	mg kg^−1^	mg kg^−1^
F_CK	8.34 ± 0.10a	9.54 ± 0.57c	2.74 ± 0.09b	98.00 ± 2.86c	27.74 ± 3.24c	255.25 ± 5.38d
F_S	7.80 ± 0.05c	14.87 ± 1.13a	4.60 ± 0.42a	132.13 ± 5.98b	19.98 ± 1.78d	560.50 ± 8.19a
F_SBC	8.23 ± 0.14ab	16.38 ± 1.89a	2.65 ± 0.33b	98.00 ± 4.95c	28.02 ± 3.18c	536.00 ± 33.51a
F_M	8.18 ± 0.07b	12.50 ± 1.43b	4.56 ± 0.70a	141.75 ± 2.02a	110.50 ± 8.16a	455.25 ± 20.71b
F_MBC	8.34 ± 0.08a	11.84 ± 1.10b	3.80 ± 0.94a	97.13 ± 8.27c	59.11 ± 6.03b	375.50 ± 12.48c
R_CK	4.53 ± 0.05d	12.64 ± 0.62c	2.76 ± 0.07c	161.88 ± 8.75a	23.55 ± 2.41c	135.75 ± 13.07d
R_S	4.51 ± 0.19d	19.31 ± 1.81a	4.79 ± 0.76a	135.63 ± 8.75b	23.36 ± 1.42c	587.00 ± 10.49a
R_SBC	4.96 ± 0.08b	16.97 ± 0.40b	2.84 ± 0.12c	130.38 ± 7.76bc	21.61 ± 1.01c	505.25 ± 8.54b
R_M	4.75 ± 0.09c	16.65 ± 1.18b	3.52 ± 0.43b	167.13 ± 8.75a	61.28 ± 2.32a	279.00 ± 39.76c
R_MBC	5.72 ± 0.05a	16.26 ± 0.69b	2.81 ± 0.45c	117.25 ± 12.94c	49.95 ± 1.73b	280.50 ± 23.30c

TOC, total organic carbon; POXC, permanganate oxidizable carbon; AN, available nitrogen; AP, available phosphorus; AK, available potassium. Data in the table are represented by mean ± SD (*n* = 4). For each soil, within a column, the different lowercase letters represent significant differences (*p* < 0.05).

Soil basal respiration and enzyme activities under different treatments were listed in Table 2. In the two soils, the SBR of S, M and MBC was higher than that of the CK, and the greatest increase was observed at S treatment. Compared with CK, F_S and F_M in the fluvo-aquic soil and R_S, R_M, and R_MBC in the red soil could increase the UA activity, and F_S in the fluvo-aquic soil and all the treatments in the red soil significantly increased the SU activity. F_MBC in the fluvo-aquic soil and R_SBC in the red soil had no significant effect, the other treatments could significantly increase the soil CA activity versus CK. In addition, compared with CK, S and M could significantly increase while SBC significantly decreased the GLU activity in the two soils, and the effect of MBC on the GLU activity in the two soils was opposite, decreasing in the fluvo-aquic soil and increasing in the red soil (Table 2). Finally, the geometric mean of enzyme activities (GMea) of the assayed enzyme activities increased 26.4 and 21.0% by S and M, and decreased 4.8% by SBC in the fluvo-aquic soil, while increased 215.9, 30.0, 283.1, and 66.2% by S, SBC, M and MBC in the red soil (Table 2).

**Table 2:** Soil basal respiration and enzyme activities in different treatments.

Treatment	SBR	UA	SU	CA	GLU	GMea
	mg kg^−1^ 24 h^−1^	mg g^−1^ 24 h^−1^	mg g^−1^ 24 h^−1^	ml g^−1^	ug g^−1^ h^−1^	
F_CK	34.98 ± 5.38c	2.78 ± 0.16b	26.80 ± 0.90bc	4.46 ± 0.24c	93.45 ± 3.62d	13.26 ± 0.43c
F_S	84.05 ± 20.82a	3.86 ± 0.18a	28.50 ± 0.08a	5.31 ± 0.10a	135.73 ± 2.83a	16.77 ± 0.21a
F_SBC	30.50 ± 1.82c	2.71 ± 0.03b	25.35 ± 1.46c	4.91 ± 0.17b	75.61 ± 2.05e	12.63 ± 0.17d
F_M	59.30 ± 8.54b	3.87 ± 0.15a	28.26 ± 0.37ab	5.21 ± 0.10a	116.55 ± 2.95b	16.05 ± 0.14b
F_MBC	45.70 ± 11.70bc	2.73 ± 0.08b	26.40 ± 1.55c	4.53 ± 0.22c	104.31 ± 6.02c	13.57 ± 0.40c
R_CK	24.45 ± 3.38c	0.34 ± 0.03d	5.75 ± 0.38c	1.01 ± 0.17d	27.47 ± 2.43c	2.71 ± 0.15e
R_S	62.63 ± 5.90a	1.17 ± 0.19b	15.75 ± 3.29a	2.21 ± 0.05c	134.44 ± 4.03a	8.55 ± 0.70b
R_SBC	20.50 ± 4.40c	0.44 ± 0.06d	14.62 ± 0.61ab	1.06 ± 0.13d	22.78 ± 1.85d	3.52 ± 0.25d
R_M	35.25 ± 6.16b	2.44 ± 0.05a	12.98 ± 1.00b	4.33 ± 0.24a	84.73 ± 2.73b	10.37 ± 0.08a
R_MBC	33.40 ± 3.60b	0.60 ± 0.05c	12.16 ± 0.86b	2.61 ± 0.45b	21.76 ± 0.83d	4.50 ± 0.32c

SBR, soil basal respiration; UA, urease; SU, sucrose; CA, catalase; GLU, β-glucosidase; GMea, geometric mean of enzyme activities. Data in the table are represented by mean ± SD (*n* = 4). For each soil, within a column, the different lowercase letters represent significant differences (*p* < 0.05).

### 3.3. Alpha and beta diversities of bacteria

**Figure 3:**
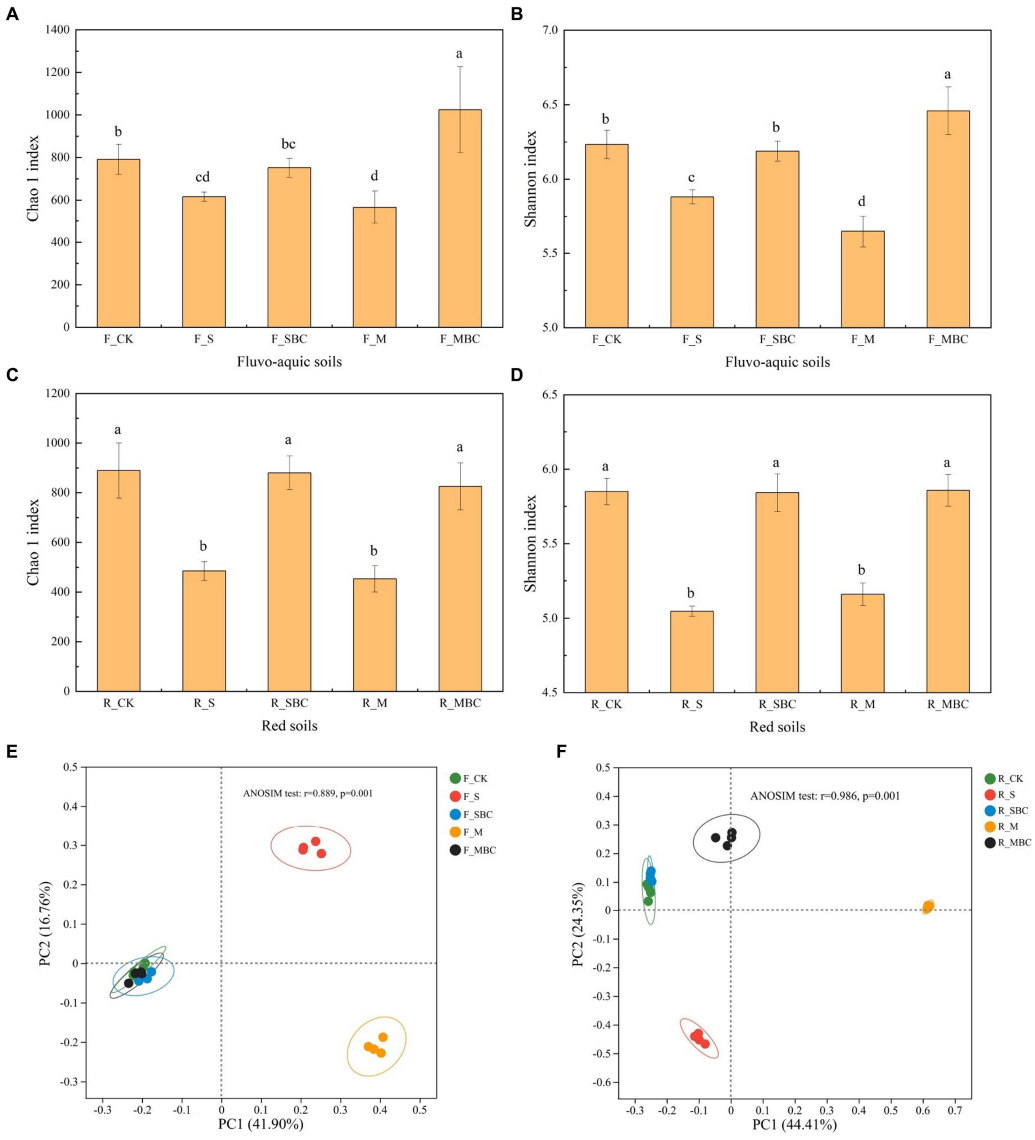
ASV-based diversity indices of bacterial community in fluvo-aquic soil **(A,B)** and red soil **(C,D)** and ASV-based PCoA analysis of bacterial community structure in fluvo-aquic soil **(E)** and red soil **(F)**. Data in the figure are represented by mean ± SD (*n* = 4). Error bars indicate standard deviation. The different lowercase letters on error bars represent significant differences (*p* < 0.05).

For the bacterial α diversity, both S and M significantly reduced the bacterial Chao1 index and Shannon index of the two soils, while SBC had no significant effect on the bacterial Chao1 index and Shannon index in the two soils. MBC had no significant effect in the red soil but significantly increased the bacterial Chao1 index and Shannon index in the fluvo-aquic soil (Figures 3A–D).

The PCoA analysis displayed that the application of different organic materials affected the different distributions of bacterial communities in the two soils. The first two principal components, PC1, and PC2 accounted for 41.9 and 16.76% in fluvo-aquic soil and 44.41 and 24.35% in the red soil, respectively. The ANOSIM analysis depicted that in the fluvo-aquic soil, F_SBC and F_MBC were closely clustered with F_CK, while F_S and F_M were separated from F_CK. In the red soil, R_SBC and R_CK were also closely clustered, R_MBC, R_S and R_M were separated from R_CK (Figures 3E,F).

### 3.4. Bacterial community members

**Figure 4:**
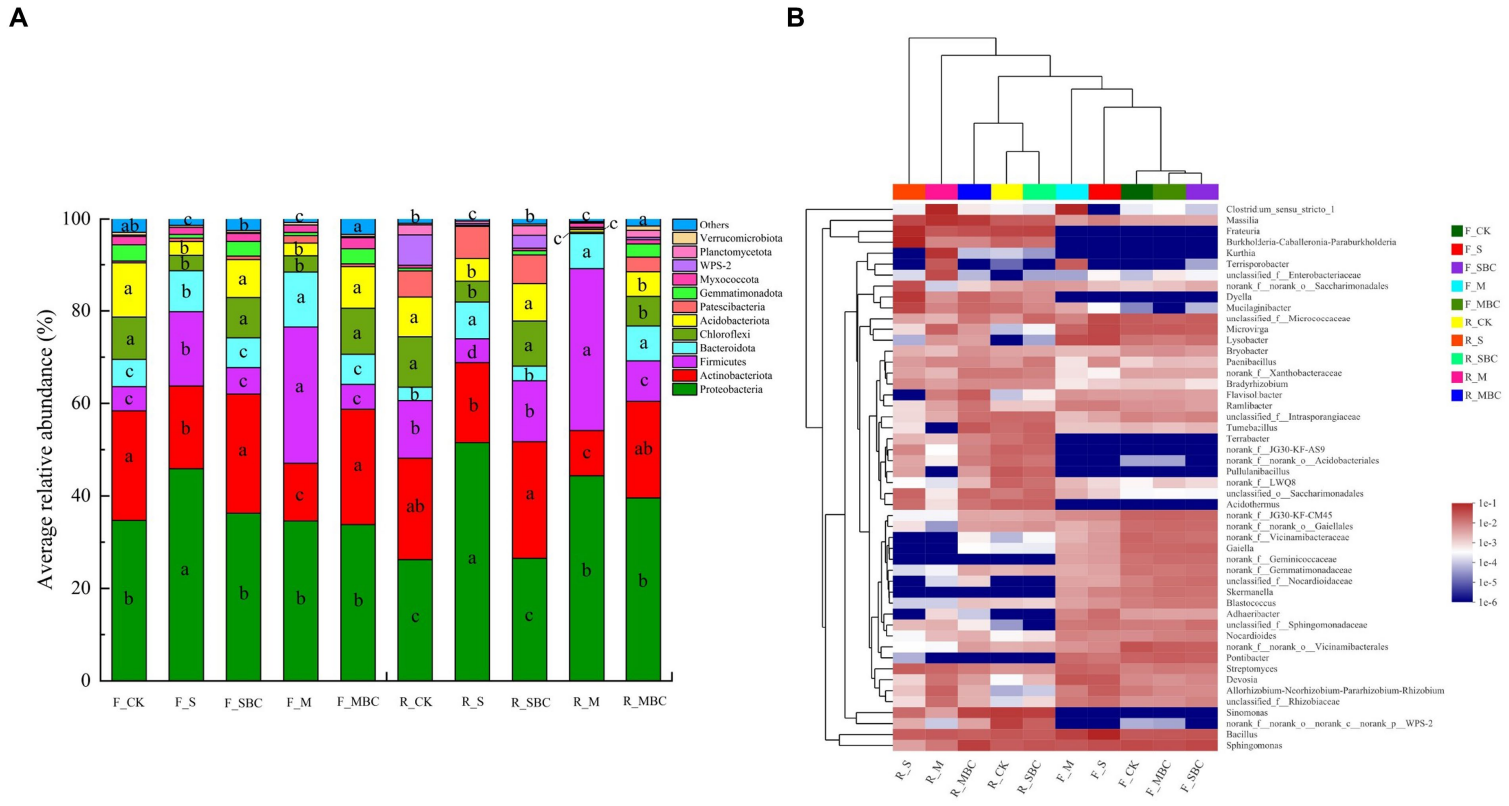
Relative abundance (>1%) of the major phyla of bacterial communities **(A)** and heatmap of differential relative abundances of microbial genera (top 50) **(B)** for different treatments. The different lowercase letters represent significant differences (*p* < 0.05).

Across all the tested soil samples, there were 12 bacterial phyla with average relative abundance exceeding 1%, and these taxa altogether accounted for more than 95% of the total bacterial recovered sequences. Among them, Proteobacteria, Actinobacteriota, and Firmicutes were the first three dominant communities, with an average relative abundance of 21.2–51.5%, 9.8–25.8%, and 5.2–35%, respectively. First, F_S significantly increased the abundance of Proteobacteria in the fluvo-aquic soil, while R_S, R_M, and R_MBC significantly increased in red soil. Second, F_S and F_M significantly decreased the abundance of Actinobacteriota in the fluvo-aquic soil, while R_M was significantly reduced in the red soil. Furthermore, S significantly increased the abundance of Firmicutes in the fluvo-aquic soil and reduced in the red soil; M significantly increased in the two soils, and R_MBC significantly decreased in the red soil (Figure 4A).

At the genus level, the bacteria genera with the top 50 abundances were clustered and presented in a heat map for ease of visualization (Figure 4B). The top 20 genera had average relative abundances in all treatment more than 1.0%, accounting for 39.33% in total. Among them, Bacillus (belonging to Firmicutes), Sphingomonas (belonging to Proteobacteria), Massilia (belonging to Proteobacteria), Frateuria (belonging to Proteobacteria) and Clostridium_sensu_stricto_1 (belonging to Actinobacteriota) were the five most dominant genera. Across the board, the relative abundance of Massilia and Frateuria were higher in red soil compared to fluvo-aquic soil, and were significantly increased by M and S treatment, respectively. The relative abundance of Clostridium_sensu_stricto_1 was increased by M treatment in two soils. The clustering indicates that, in the fluvo-aquic soil, F_SBC and F_MBC were clustered together with F_CK, which indicated that their categories were similar at genus level, but F_S and F_M were considerable different from them. Similar results were observed in the red soil, two types of biochar treatments and control were generally clustered together, which was different from the two types of biomass material treatments (Figure 4B).

### 3.5. Relationship between soil microbial community structure, soil enzyme activities and soil properties

**Figure 5:**
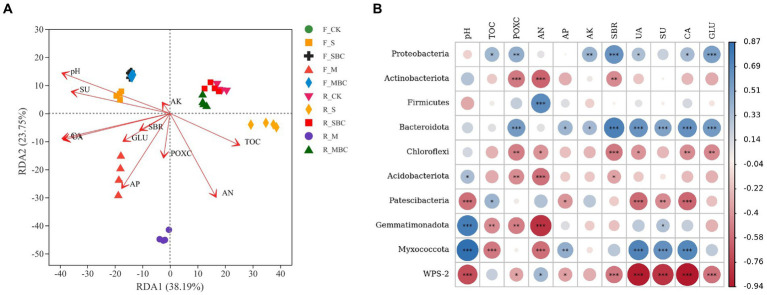
ASV-based db-RDA analysis of the relationship between bacterial communities at the genus level and soil environmental factors **(A)** and correlations between soil properties and dominant bacterial phyla **(B)**. TOC, total organic carbon; POXC, permanganate oxidizable carbon; AN, available nitrogen; AP, available phosphorus; AK, available potassium; SBR, soil basal respiration; UA, urease; SU, sucrose; CA, catalase; GLU, β-glucosidase. *p < 0.05; **p < 0.01; ***p < 0.001.

The RDA analysis revealed the relationship between the bacterial community and the soil physicochemical properties, which sufficiently explained the influence of the environmental factors on the change in the bacterial community structure at the genus level. The first axis and the second axis explained 38.19 and 23.75% of the total variance, respectively (Figure 5A). According to the Mantel test (Supplementary Table S2), pH, TOC, AN, AP, UA, SU, CA, and GLU significantly influenced the bacterial community structure in the two soils.

The correlation analysis demonstrated that *Proteobacteria*, the first dominant phylum, was positively correlated with almost all the environmental factors, among which SBR and GLU had the highest correlation, all of which reached an extremely significant level (*p* < 0.001); POXC and AK (*p* < 0.01); and TOC, UA, and CA (*p* < 0.05). *Actinobacteriota* had a high correlation with environmental factors pH, POXC, AN, and SBR, among which there was a significant positive correlation with pH (*p* < 0.05), an extremely significant negative correlation with POXC and AN (*p* < 0.001), and an extremely significant negative correlation with SBR (*p* < 0.01). In contrast to *Actinobacteriota*, *Firmicutes* was only negatively correlated with the pH (*p* < 0.05) and positively correlated with AN (*p* < 0.001), and both of the two bacterial phyla had the highest correlation with AN (Figure 5B).

**Figure 6:**
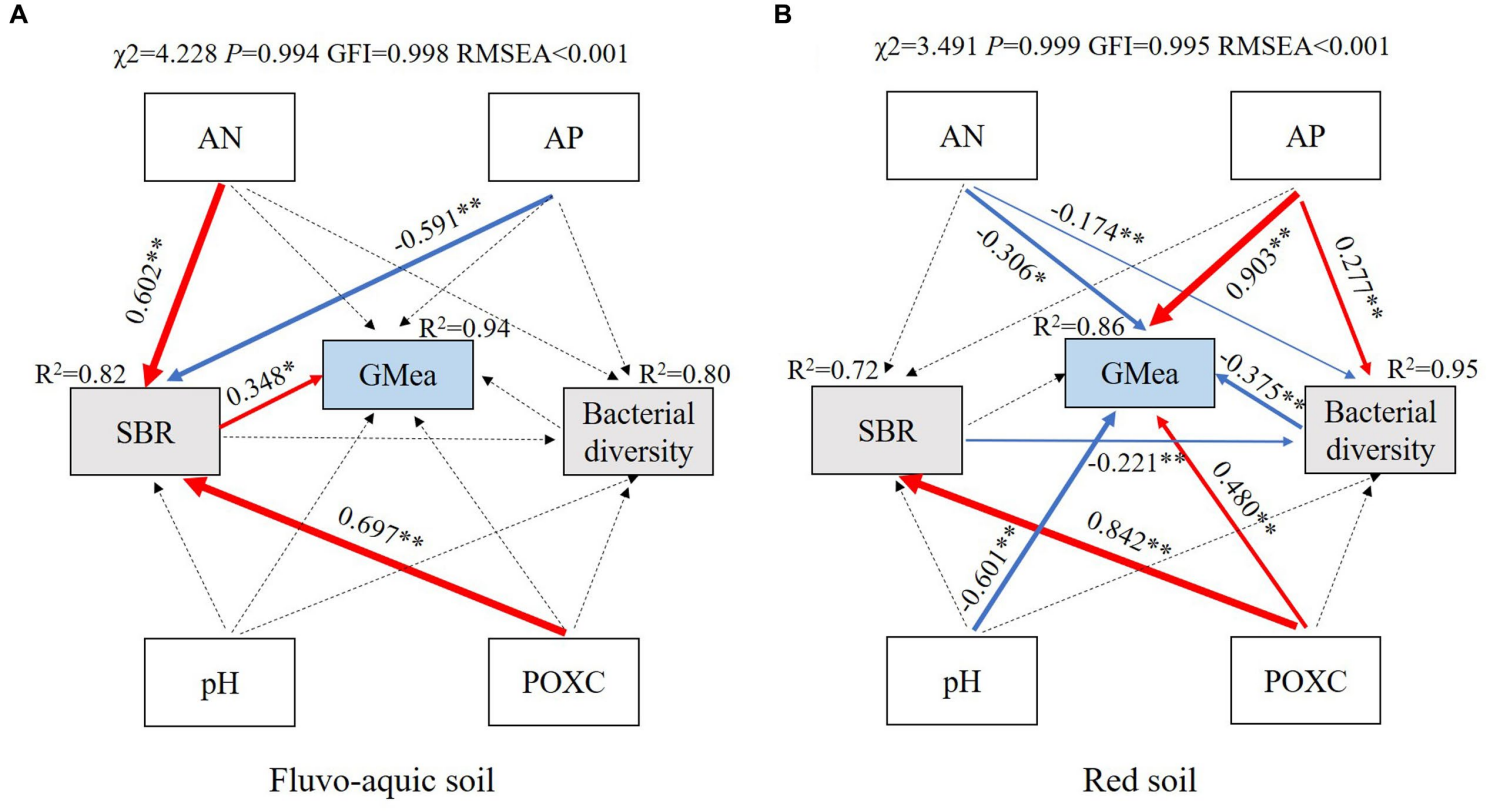
Structural equation models showing the direct and indirect effects of soil physiochemical properties, soil basal respiration, bacterial diversity and soil geometric mean of enzyme activities in fluvo-aquic soil **(A)** and red soil **(B)**. Solid and dashed arrows indicate significant and nonsignificant relationships, and red and blue arrows indicate positive and negative correlations, respectively. Numbers adjacent to the arrows are path coefficients, and the width of the arrows is proportional to the strength of the path coefficients. Significance levels are indicated: *p < 0.05; **p < 0.01. POXC, permanganate oxidizable carbon; AN, available nitrogen; AP, available phosphorus; SBR, soil basal respiration; Bacterial diversity, bacterial Shannon index; GMea, geometric mean of enzyme activities.

Structural equation models (SEMs) provided good fits to the data, and explained 94 and 86% of the variance in GMea in fluvo-aquic soil and red soil, respectively (Figure 6). AN, AP and POXC had indirect effects on GMea by changing SBR in fluvo-aquic soil, among which, AN and POXC strongly and positively contributed to SBR, whereas AP had the opposite effect. However, in red soil, pH, AN, AP and POXC exhibited direct effects on GMea, AP and POXC positively contributed to GMea, while pH and AN showed negative contribution. Moreover, POXC strongly and positively contributed to SBR, and AP positively contributed to soil bacterial diversity in red soil.

## 4. Discussion

As is well known, organic amendments can improve soil fertility, promote soil organic carbon storage and stability, and enhance crop growth (Zhu et al., 2015). In this study, the shoot biomass of maize was increased by straw-derived biochar, manure, and manure-derived biochar (Figure 1). In parallel, the potassium accumulation in shoots were increased by straw-derived biochar, while shoot nitrogen, phosphorus, and potassium accumulation were all increased by manure and its biochar in both soils. Notably, our results showed that straw-derived biochar had more significant improving effect on maize shoot biomass and nutrient resorption compared with straw in the two soils due to the significant promotion on root growth (Figures 1, 2), because of root is important organs for plant fitness and are responsible for the absorption of water and nutrients. The promoting effect of straw biochar on root growth were consistent with the findings of previous studies (Tan et al., 2017; Liu X. Y. et al., 2021). Both manure and its biochar could increase the shoot biomass in two soils and exhibited superiority over straw and its biochar owing to the higher contents of mineral nitrogen and phosphorus and the enhanced soil nitrogen and phosphorus supply and plant nitrogen and phosphorus accumulation (Table 1; Figures 1, 2). Therefore, it is apparent that the effects of the organic amendments to the soil are derived directly from its nutrient content in the short term, especially the plant bioavailable nutrients. A systematic review also found that positive yield increases were generally associated with the nutrient contents of the biochar particles, and manure biochars with high nitrogen and phosphorus contents displayed excellent yield-increasing effect (Spokas et al., 2012). Predictably, for different feedstock type biochars, manure-derived biochar with higher nitrogen and phosphorus concentrations showed more pronounced promoting effect on maize growth during the early growing stage (Figure 1). An unexpected observation was that the shoot biomass of maize was decreased by straw compared with the control, although straw had a positive effect on improving the total organic carbon, permanganate-oxidizable carbon, available potassium, basal respiration and enzyme activities in two soils. We speculate that the large quantities straw input decreased the contact area between roots and soil in the early stage, in turn, reduced the water and nutrient uptake efficiency of the plants. Morris et al. (2009) also found that placing seed with or near to straw residue could cause a restriction in crop establishment. Apparently, further studies are warranted to explore such phenomenon.

The pH of soils has generally been found to increase following biochar application, particularly in acidic soils (Zhang et al., 2015; Wu et al., 2020). Our experiments revealed that the addition of the two biochars did not significantly alter the pH of the fluvo-aquic soil, which was related to the higher pH of the fluvo-aquic soil itself. Furthermore, manure-derived biochar increased soil pH more than straw-derived biochar in the red soil because biochar was alkaline after anaerobic pyrolysis, and manure contained more alkaline components than straw (Siedt et al., 2021). Multiple studies had shown that the effect of biochar on the nutrient availability in soils were depended on soil and biochar types (Brassard et al., 2016; Jin et al., 2016). In this study, we found that two types of biochar reduced the content of available nitrogen in red soil significantly, but had no significant effect in fluvo-aquic soil (Table 1). These findings are consistent with other previously published trials (Zhang et al., 2015; Wu et al., 2020). Nitrification is essential step in the soil nitrogen cycle, which has an optimal point at pH 8.0 and does not occur at a pH lower than 5.5 (Sahrawat, 2008), therefore the NH_4_^+^-N is generally the dominant nitrogen form in acid red soil. Based on the above analysis, the available nitrogen content in red soil decreased with biochar amendment, possibly because biochar with abundant oxygen-containing functional groups and high porous showed better adsorption capacities on NH_4_^+^-N compared to NO_3_^−^-N. Additionally, direct nutrient input may be the main reason for biochar to affect soil phosphorus and potassium supply, we found manure and its biochar with higher phosphorus concentrations had a more ameliorating effect on soil available phosphorus, whereas straw and its biochar with higher potassium concentrations exhibited a better effect on increasing available potassium (Supplementary Table S1; Table 1). Furthermore, four organic materials significantly increased the total organic carbon of the two soils, while straw and manure significantly increased the permanganate-oxidizable carbon content and soil basal respiration in two soils rather than their biochars. It has been demonstrated that a positive relationship exists between soil basal respiration and soil permanganate-oxidizable carbon content (Zhang et al., 2021). This can be attributed to the fact that the easily oxidized organic carbon in straw and manure is utilized by the soil microorganism as the energy source and emitted carbon dioxide in the short term, while the input by biochar is predominantly stable carbon that is resistant to decay (Zhao et al., 2020). In short, straw and manure showed greater effect on improving POXC content and basal respiration compared with their derived biochar in both fluvo-aquic and red soil.

Soil enzymes, as bioactive indicators for evaluating soil quality, are often affected by soil physiochemical properties and microorganisms (Ghosh et al., 2020). In the present study, we found straw and manure showed more significant promoting effect on soil enzyme activities compared with their biochar in both fluvo-aquic soil and red soil (Table 2). This may be attributed to the input of easily oxidized organic carbon in straw and manure as demonstrated previously, which could quickly modulate the microbial community composition and change soil enzymes during a short-term experiment (Wei et al., 2019). To test this hypothesis, structural equation models were performed in two soils. The results showed that soil basal respiration, which showed strong positive connection with soil available nitrogen and easily oxidized organic carbon, was great predictor for soil enzyme activities in fluvo-aquic soil, while soil available phosphorus and easily oxidized organic carbon were positive regulator of soil enzyme activities in red soil (Figure 6). This may explain why the soil treated by manure derived biochar showed higher soil geometric mean of enzyme activities compared with straw derived biochar in both fluvo-aquic soil and red soil.

The results of alpha diversity indicated that straw and manure significantly reduced the Chao1 and Shannon index in both fluvo-aquic and red soil (Figure 3), which was different from previous studies (Zhao S. C. et al., 2019; Li et al., 2021). The results could be inconsistent because ours was a short-term experiment, soil microbes were sensitive to external additives, and straw and manure imported large amounts of labile organic carbons into the soil which created a relatively easy-to-use nutrient environment for the soil microbes. Thus, some bacteria with a high utilization efficiency of labile organic carbons grew faster, in turn, inhibiting the growth of the other bacteria in a short period. Our experiment on the main bacterial phylum also proved that straw and manure significantly increased the relative abundances of Proteobacteria, Firmicutes, and Bacteroidota in the two soils, and they occupied three of the top four dominant phyla. Therefore, the niche of the other phyla was severely compressed (Figure 4A). The same study also found straw and manure reduced the complexity of the soil bacterial co-occurrence networks, which is a key component of bacterial biodiversity (Liu et al., 2020; Ge et al., 2021; Chen et al., 2022). In addition, manure-derived biochar significantly increased the Chao1 index and the Shannon index of bacteria rather than straw-derived biochar in the fluvo-aquic soil (Figures 3A,B). This can be attributed to the more reasonable carbon-to-nitrogen ratio and richer nutrients in manure-derived biochar (Supplementary Table S1), which could significantly increase the POXC content of the fluvo-aquic soil (Wu et al., 2021; Table 1). Additionally, soil available phosphorus was another key factor that affected the diversity of soil bacteria (Chen et al., 2017), and manure-derived biochar enhanced available phosphorus of the fluvo-aquic soil more considerably (Table 1). In summary, as expected, straw and manure showed more pronounced short-term effects on soil bacterial diversity and community structure compared with their derived biochars.

As is well known, soil environment variations are the principal driving forces of changes in microbial diversity and community compositions in soil. Members of different communities prefer different ecological niches. Organic materials and their derived biochars affect soil microbial community primarily by altering the soil physicochemical properties in the short term. The RDA results revealed that soil properties contributed to over 60% of the alterations in the composition of the bacterial community (Figure 5A), suggesting that these soil environmental factors played a dominant role in the construction of the microbial community structure. Furthermore, soil bacterial community composition exhibited different responses to straw and manure addition, with more marked effect of straw on the relative abundance of Proteobacteria and of manure on Firmicutes at the phylum level (Figure 4A). Zhang et al. (2021) also found the significantly increasing effect of straw retention on the relative abundance of Proteobacteria, which could be because many members of phylum Proteobacteria are important saprophytes capable of decomposing plant debris and more efficiently utilized cellulose as a carbon source, such as the genus Bradyrhizobium (Ge et al., 2021). We also found the abundance of Proteobacteria showed significant positive correlation with soil basal respiration and β-glucosidase, which were increased by straw addition significantly in two soils (Table 2; Figure 5B). Moreover, soil N availability favored the growth of Firmicutes, which is a dominant diazotrophic phyla (Wang et al., 2022). Therefore, manure increased the abundance of Firmicutes due to its best improving effect on soil available N. The positive correlation between Firmicutes abundance and soil available N could also demonstrate the above speculations (Table 2; Figure 5B). For two biochars, there was no significant difference between straw-derived biochar and control, while manure-derived biochar could altered bacterial community composition in the red soil, by increasing the relative abundance of Proteobacteria and Bacteroidota, and decreasing that of Firmicutes, the increased soil pH, basal respiration, enzyme activities and decreased soil available N are likely to be one of the main reasons, the results of correlation analysis can support these suggestions (Figure 5B). Some studies have displayed that Proteobacteria are significantly regulated by the soil nutrient indicators, and Firmicutes are generally positively correlated with the soil AN and negatively correlated with the soil pH (Lu et al., 2020; Muneer et al., 2022).

## 5. Conclusion

This study explored the short-term effects of two organic materials and their derived biochars on maize growth, soil properties, and microbial community structure in fluvo-aquic and red soil. Our research demonstrated that straw-derived biochar is more effective than straw in improving maize shoot biomass and nutrient resorption, because of its significant promotion on root growth. For manure and its biochar, although both exerted a positive effect on maize shoot biomass, manure-derived biochar amendments showed superiority over manure in the fluvo-aquic soil, the opposite situation was observed in the red soil. In addition, due to the input of the labile organic carbons, straw and manure showed more pronounced short-term effects on soil basal respiration, enzyme activity and bacterial community structure versus their derived biochar. In summary, in our opinion, straw-derived biochar had more obvious advantage than straw in promoting maize growth and nutrient resorption at seedling stage, while the choice of manure or its biochar should be determined by the soil type.

## Data availability statement

The datasets presented in this study can be found in online repositories. The names of the repository/repositories and accession number(s) can be found in the article/Supplementary material.

## Author contributions

YZ and XY conceived the research, performed the experiments, and analyzed the data. YZ, XY, XL, FW, and CS wrote and edited the manuscript. All authors contributed to the article and approved the submitted version.

## Conflict of interest

The authors declare that the research was conducted in the absence of any commercial or financial relationships that could be construed as a potential conflict of interest.

## Publisher’s note

All claims expressed in this article are solely those of the authors and do not necessarily represent those of their affiliated organizations, or those of the publisher, the editors and the reviewers. Any product that may be evaluated in this article, or claim that may be made by its manufacturer, is not guaranteed or endorsed by the publisher.

## Footnotes

^1^ https://cloud.majorbio.com
