# Supplementary material for: Contrasting effects of organic materials versus their derived biochars on maize growth, soil properties and bacterial community in two type soils
Source: Front Microbiol. 2023 May 25;14:1174921. doi: 10.3389/fmicb.2023.1174921 (PMC10247979; doi:10.3389/fmicb.2023.1174921)
Supplement: Supplementary file 1 [file Data_Sheet_1.docx]

Supplementary Material

# Supplementary Tables

Table S1. Chemical property of the organic materials and their derived biochar

| Materials | pH | TOC | Total-N | C/N | Total-P | Total-K |
| --- | --- | --- | --- | --- | --- | --- |
|  |  | g kg^-1^ | g kg^-1^ |  | g kg^-1^ | g kg^-1^ |
| Wheat straw | -- | 397.2 | 9.5 | 41.8 | 0.8 | 14.5 |
| Wheat straw-derived biochar | 10.32 | 701.4 | 14.6 | 48 | 2.1 | 31.5 |
| Swine | -- | 321.0 | 18.6 | 17.3 | 12.4 | 10.2 |
| Swine manure-derived biochar | 10.95 | 512.5 | 21.6 | 23.7 | 33.2 | 15.4 |

Table S2. Correlations between bacterial community structure and soil properties

| Soil variables | two soils | |
| --- | --- | --- |
|  | *R^2^* | *p* value |
| pH | 0.8743 | 0.001 |
| TOC | 0.3752 | 0.001 |
| POXC | 0.1198 | 0.086 |
| AN | 0.5762 | 0.001 |
| AP | 0.4992 | 0.001 |
| AK | 0.0086 | 0.852 |
| SBR | 0.0719 | 0.233 |
| UA | 0.7976 | 0.001 |
| SU | 0.6718 | 0.001 |
| CA | 0.8095 | 0.001 |
| GLU | 0.1861 | 0.018 |

*R*^2^ and *p*-values were retrieved from the RDA on the community structures. TOC: total organic carbon, POXC: permanganate oxidizable carbon, AN: available nitrogen, AP: available phosphorus, AK: available potassium, SBR: soil basal respiration, UA: urease, SU: sucrose, CA: catalase, GLU: β-glucosidase.
